# Novel Formulations of Phase Change Materials—Epoxy Composites for Thermal Energy Storage

**DOI:** 10.3390/ma11020195

**Published:** 2018-01-26

**Authors:** Maria Elena Arce, Miguel Angel Alvarez Feijoo, Andres Suarez Garcia, Claudia C. Luhrs

**Affiliations:** 1 Defense University Center, Spanish Naval Academy, 36920 Marin, Spain; alvarezfeijoo@cud.uvigo.es (M.A.A.F.); asuarez@cud.uvigo.es (A.S.G.); 2 Mechanical and Aerospace Engineering Department, Naval Postgraduate School, 700 Dryer Rd., Watkins Hall, Monterey, CA 93943, USA

**Keywords:** PCM, PCM-epoxy composite, thermal energy storage, paraffin, Plackett-Burman

## Abstract

This research aimed to evaluate the thermal properties of new formulations of phase change materials (PCMs)-epoxy composites, containing a thickening agent and a thermally conductive phase. The composite specimens produced consisted of composites fabricated using (a) inorganic PCMs (hydrated salts), epoxy resins and aluminum particulates or (b) organic PCM (paraffin), epoxy resins, and copper particles. Differential Scanning Calorimetry (DSC) was used to analyze the thermal behavior of the samples, while hardness measurements were used to determine changes in mechanical properties at diverse PCM and conductive phase loading values. The results indicate that the epoxy matrix can act as a container for the PCM phase without hindering the heat-absorbing behavior of the PCMs employed. Organic PCMs presented reversible phase transformations over multiple cycles, an advantage that was lacking in their inorganic counterparts. The enthalpy of the organic PCM-epoxy specimens increased linearly with the PCM content in the matrix. The use of thickening agents prevented phase segregation issues and allowed the fabrication of specimens containing up to 40% PCM, a loading significantly higher than others reported. The conductive phase seemed to improve the heat transfer and the mechanical properties of the composites when present in low percentages (<10 wt %); however, given its mass, the enthalpy detected in the composites was reduced as their loading further increased. The conductive phase combination (PCM + epoxy resin + hardener + thickening agent) presents great potential as a heat-absorbing material at the temperatures employed.

## 1. Introduction

Consensus regarding the implementation of environmental policies and energy-saving practices are increasingly present at the regional, national or international level [1]. The main reasons for implementation are, on the one hand, to reduce the increasingly high cost of energy dependence on fossil fuels and, on the other hand, to reduce the polluting emissions of those fossil fuels, which are largely responsible for the greenhouse effect [1,2]. This problem has led to the development and optimization of other energy resources and new energy storage systems [3,4,5,6,7,8,9,10]. The latter present two important advantages: the reduction of the dependence on fossil fuels and the possibility of matching energy supply and demand when they do not coincide in time [11,12]. In the case of building materials, energy-saving practices can be attained by the use of thermal energy storage (TES) systems. Sensible heat storage by changing the temperature of a storage material is a very common method of energy storage since thermal energy is stored and released in a passive way. However, its main disadvantage is that it needs a large volume of material to store the same energy as other systems based on the latent heat of the phase change. Hence, an effective way to store thermal energy is by incorporating phase change materials (PCMs) in passive latent heat thermal energy storage (LHTES) systems [13,14]. PCMs are substances that absorb or release heat during a phase transformation. In these systems, PCMs have the capacity to adapt the periods of energy supply to those of demand. This creates a field of research for the improvement of energy efficiency. Other applications of PCMs include thermal protection systems and active and passive cooling of electronic devices [15].

PCM can be classified essentially into organic and inorganic materials and, in turn, into mixtures or eutectics [12]. The phase changes that can result in heat absorbed include transformations from solid to liquid, liquid to vapor, and solid to solid. The liquid-solid change tends to be preferred, given the amount of energy exchanged during the transformation and minimal volumetric changes. Volume expansion of the PCM in a matrix during the transformation has been associated with internal residual stress and decline in mechanical properties. Another advantage of these materials is that the release or storage of energy occurs at almost constant temperature. If water is taken as an example, its specific heat is 4.186 kJ/kg·°C and its latent heat of fusion or solidification is 334 kJ/kg. This means that its capacity to store energy during phase change is approximately 80 times greater than that required to increase its temperature by 1 °C in liquid state. For a long time, water was used as the PCM in large installations, although it has fallen into disuse because of the high cost of requiring secondary fluids with low evaporation temperatures [16]. Fortunately, there are multiple other substances that can be used to store energy. Some of the requirements for a substance to be used as a PCM include having melting-freezing temperatures in the range of application, great latent heat of fusion, and large thermal conductivity. In passive LHTES systems, PCMs should also have certain properties: economic, thermophysical, chemical, kinetic end environmental feasibility. Organic PCMs are classified as paraffin and non-paraffin compounds, such as fatty acids, esters, alcohols and glycols. Inorganic PCMs are classified as hydrated salts and metallic materials. An eutectic is a minimum-melting composition of two or more components (both organic and inorganic) having a melting point lower than that of each constituent [17,18,19]. For PCM energy storage applications, paraffins are very widely used materials because of the desirable properties mentioned in Table 1.

The molecular structure of paraffins, particularly the C-atom chain length (which usually ranges from 14 to 50), is the main factor influencing their properties. For example, the melting point and latent heat of fusion increase with chain length. These materials have melting temperatures between 5.5 °C and 76 °C and solid-liquid phase change enthalpy between 170 J/g and 269 J/g (very high for organic materials). Melting paraffin has a relatively low viscosity, so it must be kept in a closed tank or container to prevent leaching [19].

The use of PCMs that change from the solid to the liquid state has a drawback: their leakage in the melting state. This reduces the heat transfer efficiency and increases production costs. To solve this problem, different solutions can be chosen. Numerous lines of research focus on microencapsulation, which consists of coating the PCM with a material that does not interact with the matrix or PCM [30,31,32,33,34,35]. Another solution being investigated is to introduce the PCM directly into the material of the matrix, without the coating material, so that the matrix would serve as the capsule of the PCM. An example of the latter is found in [36]; however, the number of studies using such an approach is very limited. Another disadvantage noted in the organic PCMs is their low thermal conductivity, which significantly affects the rates of charge and discharge of energy.

In the present study, aiming to solve the leakage problems, we selected the use of a polymer matrix—an epoxy resin—as the support material in which the PCM was to be dispersed. Composites using epoxy resin as a matrix are widely used as polymer coatings due to their good corrosion resistance, reduced shrinkage during curing, high adhesion and dimensional stability [37,38]. As previously mentioned, the literature concerning the use of epoxy resin-PCM systems is extremely scarce; the only significant study on the topic is a study that uses polyethylene glycol as PCM, epoxy as matrix and a single loading value [36]. The present effort also attempted to address the thermal conductivity drawback by including a highly conductive phase in the composites. Aluminum or copper (Cu) particles were employed as conductive phases with the expectation that their inclusion would increase the thermal conductivity of the epoxy matrix and thus increase the overall heat flow values of the composites. Three different inorganic compounds were used as the inorganic PCM for comparison with paraffin, which was selected as the organic PCM given its transition temperatures, cost and availability. A thickening agent was used to assure the homogeneous distribution of the PCMs and metal particles. This article focuses on studying the enthalpy characteristics of PCM-epoxy composites fabricated using diverse PCM and conductive phase loadings to determine the optimal amounts of each component needed to maximize the heat produced during the PCM phase transformation.

## 2. Materials and Methodology

The purpose of this investigation was, as mentioned above, to determine and quantify the impact that (a) diverse loadings of PCM (organic and inorganic); (b) different loadings of a conductive phase and (c) time elapsed before the addition of all components in the uncured epoxy system had on the thermal conductivity and mechanical properties of the epoxy resin composites. The same amount of thickening agent was added in all cases.

### 2.1. Materials

- *Epoxy resins*: For the inorganic PCM-epoxy trials, the resin system employed already contained conductive particles as part of the commercial product formulation. It employed the aluminum-filled epoxy casting system Resoltech^®^ 1450 T Alu 25, acquired from Castro Composites (Pontevedra, Spain).
  For the organic PCM-epoxy trials, the epoxy base Resolcoat^®^ VLSUV A (density = 1.1 g/cm^3^, viscosity at 25 °C = 1200 mPa·s) and the hardener Resolcoat^®^ VLSUV B (density = 0.95 g/cm^3^, viscosity at 25 °C = 1200 mPa·s) were supplied by Castro Composites (Pontevedra, Spain). The mechanical and physical properties of the mixture (epoxy resin + hardener) at 25 °C are: impact resistance = 18 J, viscosity = 1200 mPa s, density = 1.1 g/cm^3^ and TG of 45 °C. All materials were used as received. The mixture’s A:B ratio by weight was 100:70. The benefit of using this system was the option to acquire it as a bare resin and add diverse loadings of conductive particles as part of our experimental steps.
- *Inorganic PCM*: Given the known transition temperatures of some salts used in previous studies [39], three different inorganic PCM were used:
  - Na_2_S_2_O_3_·5H_2_O (assay 99.0–101.0%, melting temperature = 48.5 °C, relative density = 1.7)
  - Zn(NO_3_)_2_·4H_2_O (assay 96.0–103.0% (ex Zn), melting temperature = 36.4 °C, density = 2.07 g/cm^3^)
  - CH_3_COONa·3H_2_O, Acetic Acid Sodium Salt, (assay ≥ 98.0%, melting temperature = 58 °C, density = 1.45 g/cm^3^). These three laboratory reagents were supplied by Labbox Labware (Barcelona, Spain).
- *Organic PCM*: PR 5658 CH^®^ Paraffin wax (melting temperature = 56 °C–58 °C (ASTM D 938 method), maximum oil content = 0.5% (ASTM D 721 method), density at 25 °C = 0.8 g/cm^3^) was supplied by Ceras Martí (Barcelona, Spain). This paraffin wax is composed of straight chain paraffin hydrocarbons having carbon numbers predominantly in the approximate range of C20 through C50.
- *Conductive phase*: With the aim of improving mechanical and thermal properties of the composites, free-flowing fine Cu powder Amertek^®^ lab grade > 99.5% purity and 250–325 MESH (particle size) was used as an additive.
- *Thickening agent*: Garamite 1958 powdered rheology additive was used to increase storage stability and sag resistance. This additive is composed of organophilic phyllosilicates (water content <6% and specific weight 1.5–1.7 g/cm³). The optimal loading in the composites (5% of total weight of the epoxy resin + hardener mixture) was determined through a series of preliminary laboratory tests. As with other materials, this additive was acquired from Castro Composites (Pontevedra, Spain).

### 2.2. Fabrication Steps

For the inorganic PCM-epoxy composites, the fabrication process consisted of adding the PCM into the epoxy resin already loaded with conductive aluminum particulates followed by the addition of the hardener. Samples with 3% and 10% of PCM loading were fabricated. Given the simplicity of the approach and the number of samples prepared, no experimental design was necessary.

**Figure 1:**
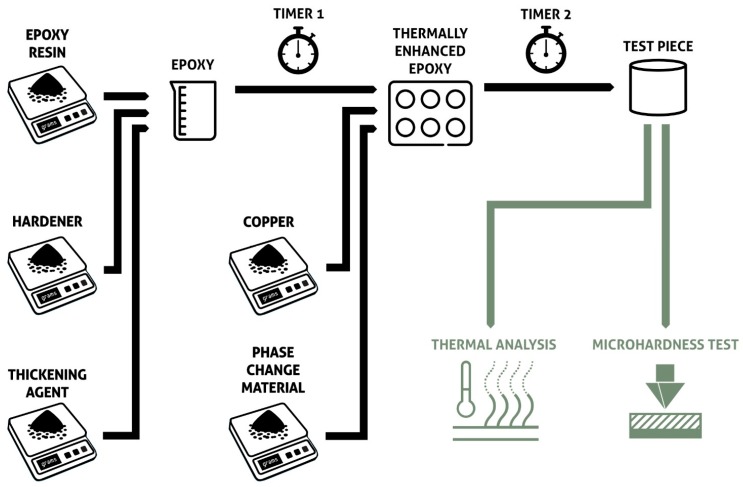
Schematic diagram of the preparation steps and techniques used for the analysis of the composites.

In contrast, the general process for the preparation and analysis of the thermally enhanced epoxy resin loaded with organic PCM is represented in Figure 1. As indicated in the figure, the epoxy resin, hardener and thickening agent (5 wt %) were thoroughly mixed before adding Cu as the conductive phase and paraffin as the organic PCM. It should be noted that all materials were used as received. That is, the PCMs were not casted before being added to the epoxy, and the epoxy resin + hardener + thickening agent composite was not heated before mixing. Two processing times were necessary: time 1 refers to the mixing time used to incorporate the epoxy resin + hardener + thickening agent (2 min for all the samples were enough to produce a homogeneous dispersion), and time 2 refers to the time elapsed before the previous step and the addition of the PCM and Cu particulates (10 to 20 min, see Section 2.3 for further information). The samples were then left to cure undisturbed for 24 h.

### 2.3. Experimental Design (Plackett-Burman) Followed for Organic PCM-Epoxy Composites

A Plackett-Burman design was used for screening the experiments that used the organic PCMs to evaluate and contrast the effect of the diverse variables in the enthalpy of the composite specimens. In order to follow an experimental design, three parameters were considered: amount of Cu, amount of PCM (paraffin), and time for paraffin and Cu addition into the epoxy mixture (time 2), and those parameters were included in a Plackett-Burman analysis. The loading values employed for Cu and paraffin were between 5% and 10 wt %, and the times selected for the PCM and Cu addition varied between 10 and 20 min. Samples were constructed with defined two-levels for each variable, a high (+) and low (−) level. Table 2 lists the set of parameters used with their low, high and zero levels. The zero level was added as the arithmetic mean of the two. The first experimental stage consisted of nine different samples. With the result of these samples, we targeted baseline compositions (no fillers added) and increased the loadings of the most promising variables. The dummy factors used for the analysis have no material or physical/chemical meaning. These dummy factors are commonly used to discern the significant factors. That is, the apparent effects of these dummy factors can be used to estimate the random measurement errors. Thus, the more dummy factors there are, the better one can estimate the random errors [40].

The results section presents the detailed experimental design matrix along with the results produced. The heat flow signal obtained was employed as criteria to evaluate the design parameters.

### 2.4. Characterization Methods

In order to examine the particle size of the copper filler used as conductive phase, some of the Cu powder was attached to a standard aluminum holder using carbon tape and introduced in the chamber of a Zeiss Neon 40 High Resolution Scanning Electron Microscope (SEM) operating at 20 kV. Secondary electron images were collected at diverse magnifications.

A Netzsch STA 449 F3 Jupiter with simultaneous TGA and DSC capabilities was employed to study the thermal properties of all the samples (±2% J/g, ±0.001 K). The specimens were exposed to an Ar/O_2_, 80%/20% atmosphere from RT to 80 °C at a heating rate of 2 °C/min. It is worth noting that in this manuscript, the area of the peaks found in DSC for individual PCM compound transformations is referred to as heat of fusion (J/g), while the peaks found in composite samples, given that not all the samples suffered a solid-liquid transformation, are referred to as enthalpy (J/g). The STA temperature and sensitivity calibrations were performed using the melting point of five different substances: gold, aluminum, indium, tin and bismuth. An empty crucible was used for buoyancy correction using the same atmosphere and heating rates as those used in the experiments.

The epoxy composites were sectioned, and a cross section of each was polished using 300, 600, 1200 and 2400 grit paper and then cleaned using a cloth to remove scratches from the sample surface. Multiple point microhardness data were then collected using a Struers DuraScan Vickers Hardness Tester (HV0.01-HV50).

## 3. Results and Discussion

### 3.1. Inorganic PCM-Epoxy Composites

**Figure 2:**
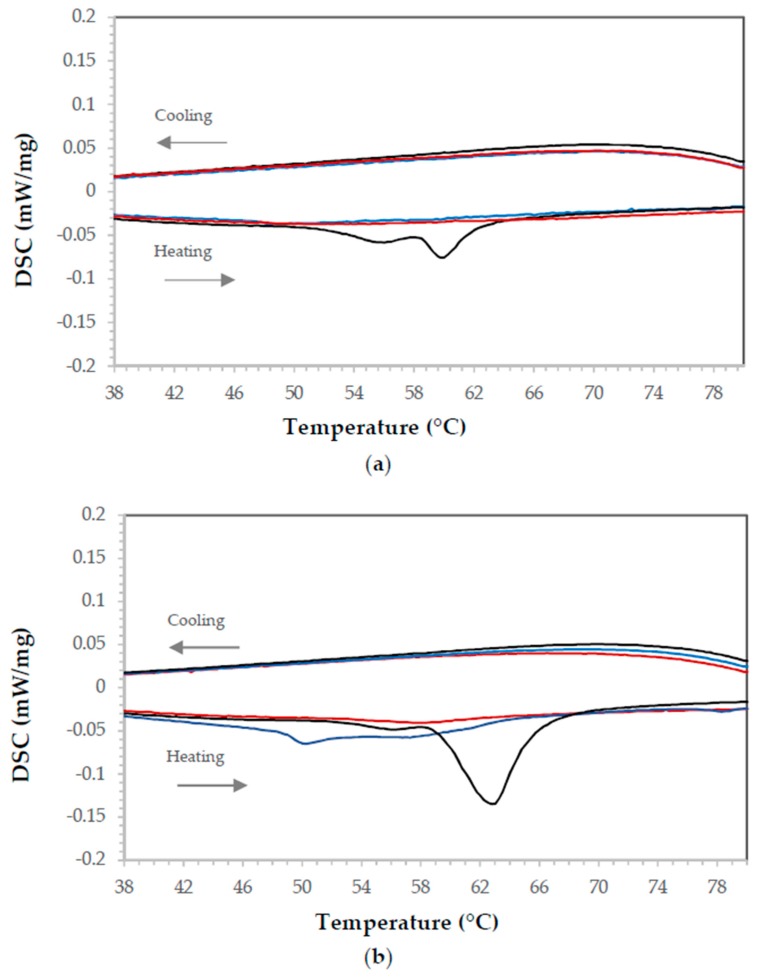
Endothermic peaks observed in Conductive Epoxy samples: (**a**) containing 3% PCM and (**b**) containing 10% PCM. PCM1, in red, corresponds to Zn(NO_3_)_2_·4H_2_O; PCM2, in blue, corresponds to Na_2_S_2_O_3_·5H_2_O; and PCM3, in black, corresponds to NaCH_3_COO·3H_2_O.

The analysis of the heat flow signal in the STA instrument was used to evaluate the effectiveness of mixing inorganic PCMs with epoxy resin containing aluminum. The results obtained are shown in Table 3. As can be seen from the data, the highest heat of fusion values corresponded to PCM3 (NaCH_3_COO·3H_2_O), which presented a peak area five times larger than the one observed for PCM1 (Zn(NO_3_)_2_·4H_2_O). The sodium sulfate pentahydrate (PCM2, Na_2_S_2_O_3_·5H_2_O) showed an intermediate behavior. The endothermic transformations for the three salts occurred at different temperatures, with peaks located at 40.5, 70.2 and 68.5 °C for the PCM1, PCM2 and PCM3 respectively. The results of heat of fusion (J/g) are similar to those shown in previous studies [41,42]. The inorganic PCMs did not to present a reversible transformation when subjected to a complete heating-cooling cycle. That is, an endothermic peak was observed during the heating cycle, but no exothermic peaks were found in the cooling stage when the salts were independently analyzed. Once the PCMs were included in the epoxy composites, the heat flow signal still showed peaks that corresponded in temperature to those encountered in the salts analysis (no epoxy included). As expected, the higher the percentage of PCM, the higher the DSC signal (see Figure 2). The lack of reversibility is presumably due to the high sub-cooling temperatures needed for these salts to completely solidify [42,43]. For instance, for the sodium acetate trihydrate (PCM3), with a melting point of 58 °C, no crystallization could be observed up to −50 °C [42]. In some cases, the different tendency to sub-cooling of the melts could be used for a specific release of heat by starting the crystallization with the addition of crystals [44], but this was not an object of study since the PCMs were embedded into a matrix (epoxy resin).

In sum, the heat flow signals of the inorganic PCM-conductive epoxy mixtures were easily detected by the instruments employed, demonstrating that the epoxy could be used as a reservoir for the PCM substance. Moreover, a mixture of these salts could render PCMs with targeted transformation temperatures. Unfortunately, given the nonreversible nature of the inorganic PCM phase transition, the study moved on to the use of organic PCM-epoxy systems known to be stable over a long range of heating and cooling cycles.

### 3.2. Organic PCM-Epoxy Composites

#### 3.2.1. Visual and Microscopic Observations of Phases

**Figure 3:**
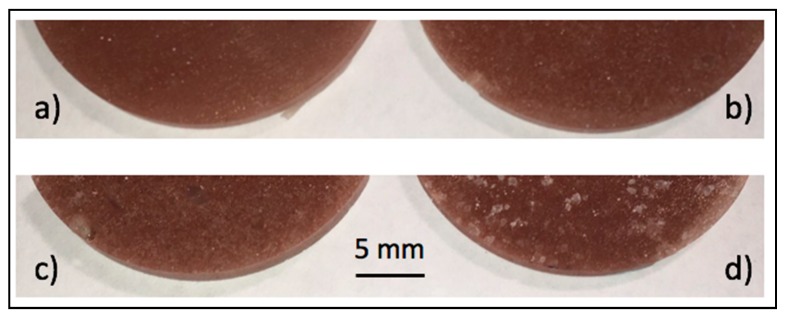
Paraffin dispersion as a function of curing time and loading. Top: Samples with 5% Cu and 5% paraffin. Fillers were added after (**a**) 10 min and (**b**) 20 min of curing time. Bottom: Samples with 10% Cu and 10% paraffin. Fillers included after (**c**) 10 min and (**d**) 20 min of curing time.

Visual observations of the organic PCM-epoxy composites phase distribution showed that samples with low levels of loading presented more homogeneous color and phase distributions (see Figure 3a,b), while higher levels of loading showed more evident phase segregation (Figure 3b,d). Addition of fillers (paraffin and Cu) after 20 min of curing time produced samples with highly inhomogeneous dispersions (see Figure 3c,d). Moreover, the visual observation analysis revealed that the PCM was packaged firmly by the epoxy matrix (epoxy resin + hardener + thickening agent). That is, in this case, the epoxy matrix acted as a capsule for the PCM, providing a mechanically robust structure to the composite and keeping the shape of the samples in the solid state. Based on low magnification observations, the paraffin and Cu were uniformly distributed throughout the epoxy matrix when their loadings did not exceed 5 wt %. It is important to highlight that without the thickening agent addition, it would not be possible to create a uniform matrix with this PCM percentage in weight. The thickening agent creates a three-dimensional network in the epoxy matrix that hinders segregation of additives. In the samples with 10 wt % of paraffin, the matrix surface presented multiple white spots, and inhomogeneous sections could be easily identified. The time elapsed between the moment when the resin, hardener and thickening agent were mixed and the PCM and Cu were added seems to have had a greater effect on the components distribution, as observed in Figure 3. Only samples produced after ten minutes had a homogeneous color.

**Figure 4:**
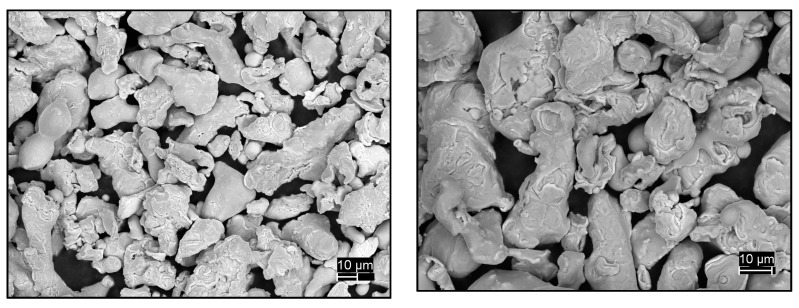
Scanning Electron Micrographs of Cu powder employed as conductive phase.

The micro-structure of the Cu filler was analyzed by a Scanning Electron Microscope (SEM), as shown in Figure 4. Secondary electron images collected at diverse magnifications revealed a particle size within the 5–70 μm range. All particles presented highly irregular shapes. According to percolation theories [45], given the morphology observed, high loadings of Cu will be required in order to produce significant changes in the samples properties.

#### 3.2.2. Thermal Analysis

##### *Paraffin*

**Figure 5:**
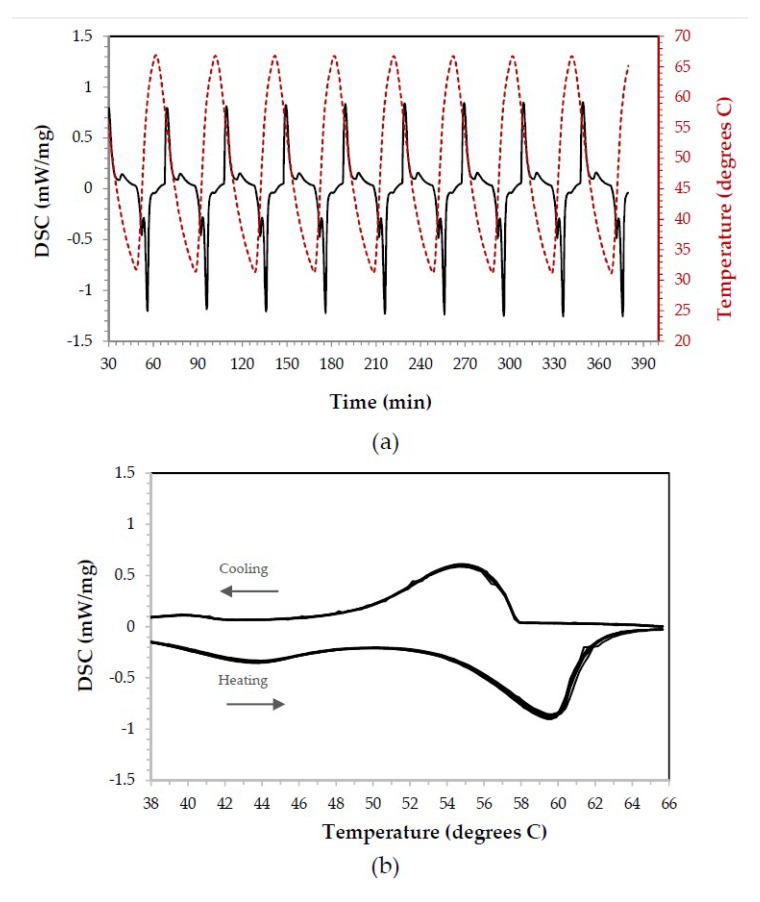
DSC results for paraffin over 8 cycles. (**a**) Performance over time, (**b**) endothermic and exothermic peaks observed during the melting and solidification processes, respectively. Exothermic peaks up, endothermic peaks down.

The paraffin without epoxy or other component added was analyzed to confirm the existence of a reversible behavior. The scan cycle was conducted as follows: heating from 25 °C to 65 °C and cooling from 65 °C to 25 °C. This temperature range was selected because it comprised the paraffin (organic PCM) phase change expected temperature. The DSC scans (Figure 5a) were repeated ten times to obtain averaged values (heat of fusion of 113.7 J/g) and to observe the reversibility of the process. Contrary to inorganic PCMs from the same range of heating and cooling cycle temperatures, endothermic and exothermic curves were observed (Figure 5b). The first melting cycle was not taken into account for calculations, an accepted practice when using this technique and even during calibration of the same.

Once it was verified that the paraffin showed reversibility in the temperature range under study, the next step was taken: identify the signal of the cured epoxy resin without any PCM or thickening agent added.

##### *Cured Epoxy Resin*

**Figure 6:**
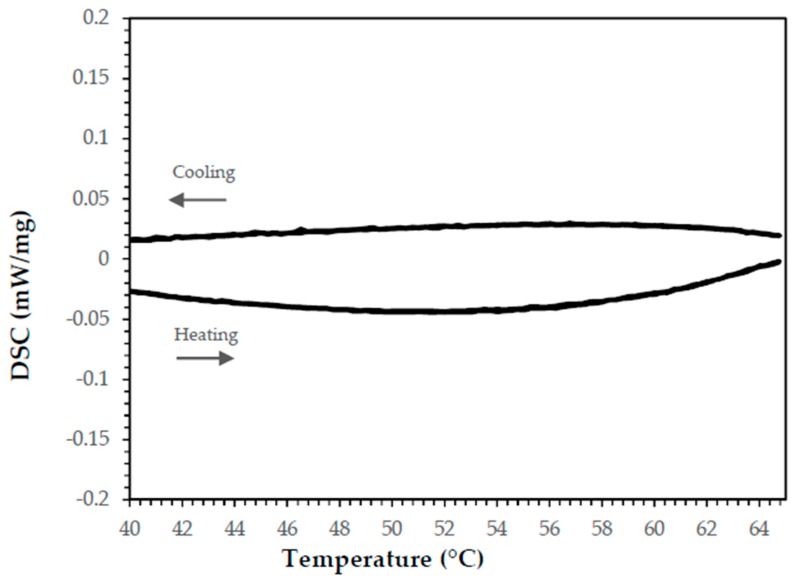
DSC results for endothermic and exothermic capacity for epoxy resin (Resolcoat^®^ VLSUV).

Figure 6 shows the epoxy resin signal, where no endothermic or exothermic peaks are present. This indicates that the effect observed in the enthalpy is exclusively due to paraffin and Cu.

##### *Organic PCM-Epoxy Composites*

**Figure 7:**
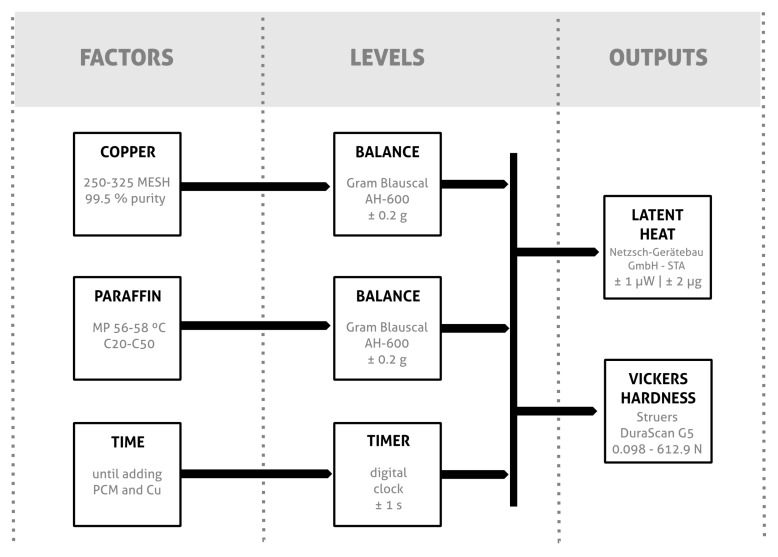
Schematic diagram of the variables analyzed and the methods used to characterize the samples.

The main approach to create test samples with the epoxy resin and the additives (PCM-paraffin and conductive phase-Cu) was, as stated in Section 2.3, the use of a design of experiments based in the Plackett-Burman method. The test pieces were made according to the flow described in Figure 7, which shows in a schematic way the factors and the methods employed to analyze the samples.

Table 4 shows the DSC results, the samples composition and the Plackett-Burman (PB) results. ANOVA-related estimations are enough to analyze the PB [40]. The three estimators of the significance of the factors (Effect, SS and *F*-value) were calculated based on previous literature [40,46,47]. The factors A, B and C may be compared using a one-tailed *F*-test (*p* = 0.05). The critical value of F_1,4_ at *p* = 0.05, was 7.71. Thus, only the effect of changing the level of factors B and C seems significant. The same approach shows that factor A (conductive phase) seems to have no considerable effect at this significance level. A significant effect was found only for the amount of paraffin and the time to wait to add paraffin and Cu. These results highlight the importance of setting the correct test type and providing a physical interpretation to such results.

**Figure 8:**
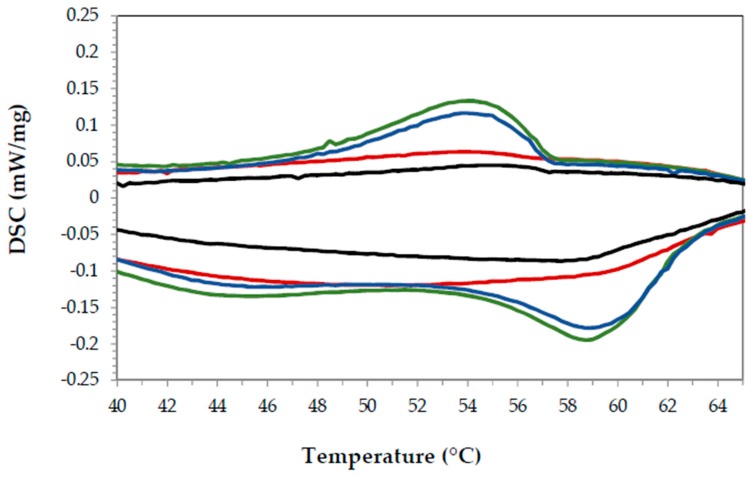
DSC results for samples 1 (red), 3 (green), 4 (blue) and 8 (black) from the Plackett-Burman experimental design for time 1 (10 min). Exothermic up, endothermic down.

In order to compare the heat flow during the PCM transformations, the samples were grouped by time elapsed to add paraffin and Cu. That is, in all these samples, the waiting time to add paraffin and Cu was ten minutes. Figure 8 shows the DSC results for samples #1, #3, #4 and #8 (see Table 4). It can be observed that for the same time (the same level of variable C), the enthalpy signal recorded by the DSC increases for higher amounts of paraffin. That is, samples 3 and 4 have larger enthalpies (during both melting and solidification) than 1 and 8, in agreement with the Plackett-Burman analysis.

However, samples 1 (red) and 3 (green), which correspond to higher amounts of Cu (10%), present higher enthalpy values than the sample containing 5% of Cu and 5% paraffin, but sample 8 (black) disagrees with that analysis. According to the mathematical outcome of the Plackett-Burman analysis, there is no significant effect in the sample enthalpy by the addition of different amounts of Cu (Table 4). However, that outcome depends on the threshold selected in the data as significant (*F*-value mentioned above) and, as can be seen in Figure 8, does not align with the observed data, as both the Cu and paraffin additions seem to have a positive effect in the DSC enthalpy results. Given that those two variables (PCM and Cu) were confounded and that the PB analysis only comprised nine samples, more samples were prepared to include higher loadings of both Cu and paraffin (listed in Table 5). All the samples prepared outside of the PB methodology employed ten minutes as timer 1 (see Figure 1) and 5% of the thickening agent. Samples with only Cu and only paraffin were also prepared.

**Figure 9:**
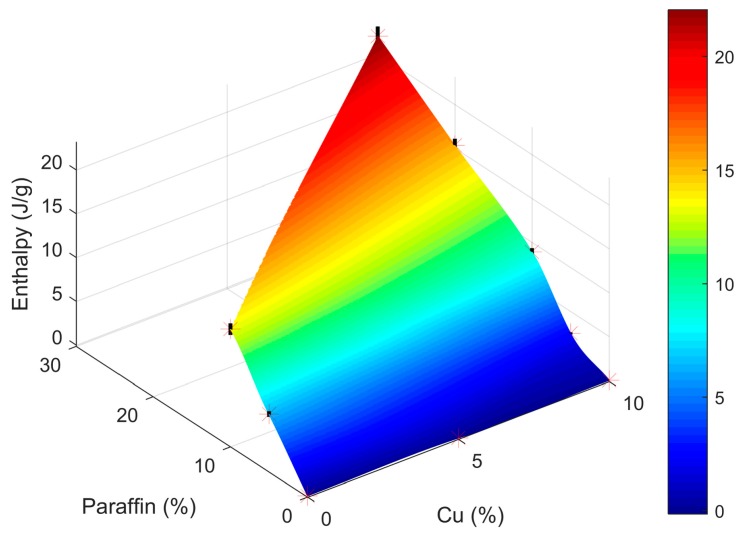
Enthalpy (J/g, vertical axis) for different composites (wt % Cu and wt % paraffin PCM on bottom plane axis).

As noted in Table 5, the weight percentage ( wt %) of Cu and PCM in the second set of samples (not contained or analyzed using Plackett-Burman) was significantly increased, up to 30 wt % of Cu and up to 40 wt % of PCM. Moreover, samples with no Cu or no PCM were also analyzed. Figure 9 presents the outcome of the samples enthalpy characterization. The point plot with mesh was created using a cubic interpolation. Here it can be seen that a greater amount of paraffin produces higher enthalpy values (up to a 40 wt % only since higher PCM contents result in samples with evident component segregation). The Cu content seems also to produce greater enthalpy values until a certain degree. Indeed, for each weight percentage of paraffin, an increase in absolute enthalpy is observed as the amount of Cu is increased. Its effect, however, is not as significant as that of the paraffin itself.

**Figure 10:**
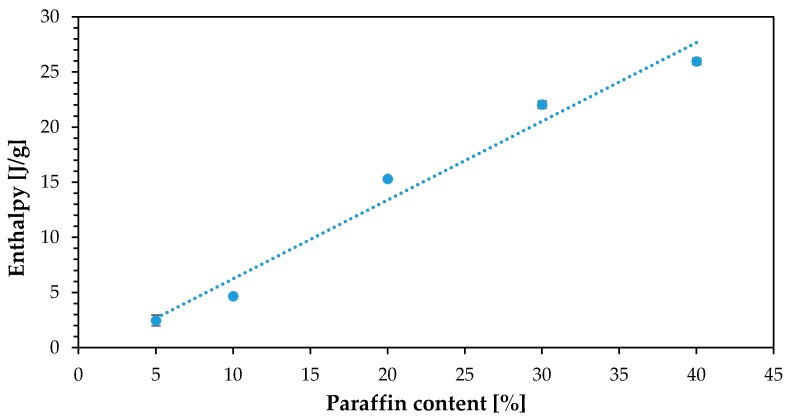
Enthalpy vs. paraffin content (all samples with 10% Cu).

Such results can be easily understood in terms of the mass that the Cu particles add to the composite; up to a 10 wt %, their effect in heat transfer is greater than their weight effect. However, for 20%, 30% and 40 wt % of Cu, the weight added reduced the overall enthalpy values since those are expressed as J/g. It is believed that for a more effective formulation, the conductive phase should be present with morphologies that will allow the particulates to contact each other with small loadings, for example using high aspect ratio fillers that will ideally also be lighter. Figure 10 shows the enthalpy tendency for different percentages of paraffin. That is, for a fixed value of Cu content, the more paraffin added, the higher the absolute enthalpy recorded for the composite.

##### *Performance over Time*

**Figure 11:**
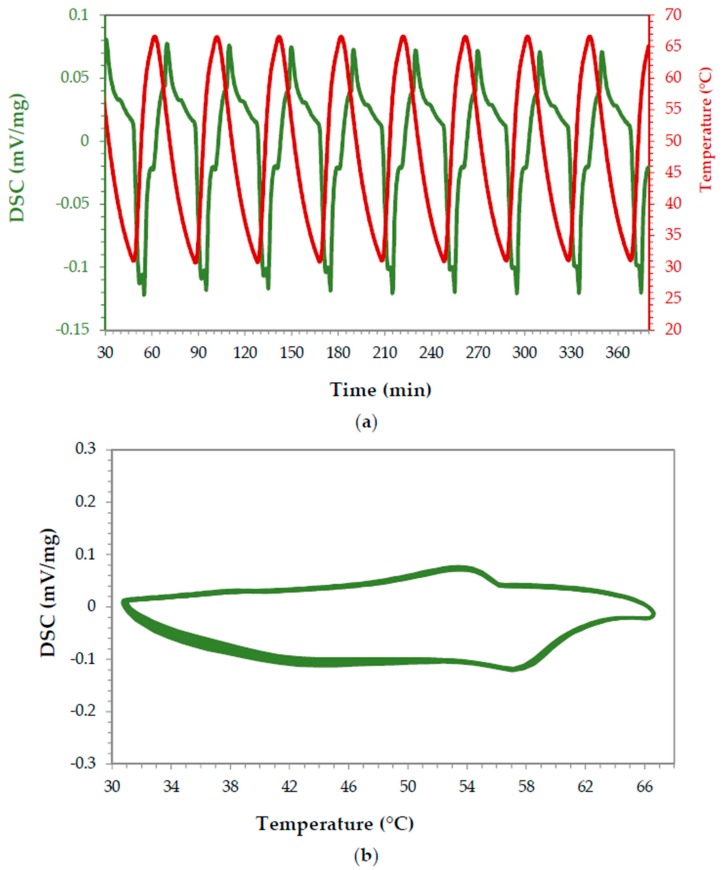
DSC results for sample #3 over 8 cycles: on the (**a**) performance over time, on the (**b**) endothermic and exothermic peaks observed as a function of temperature.

In order to study the reversibility of the PCM-epoxy composites over time, a sample containing 10% Cu and 10% paraffin that was fabricated using 10 min as time 2 was submitted to repeated heating and cooling cycles in the DSC. As in previous experiments, the temperature cycle was set between 25 °C and 65 °C. Figure 11 top shows the enthalpy results in J/g for each cycle. The average value was 10.73 J/g (STD 0.36). Figure 11 bottom shows the process reversibility over time. It was found that the sample presents the melting endothermic peak at the same temperatures and with the same areas over more than ten cycles. 

#### 3.2.3. Microhardness Test (HV).

**Figure 12:**
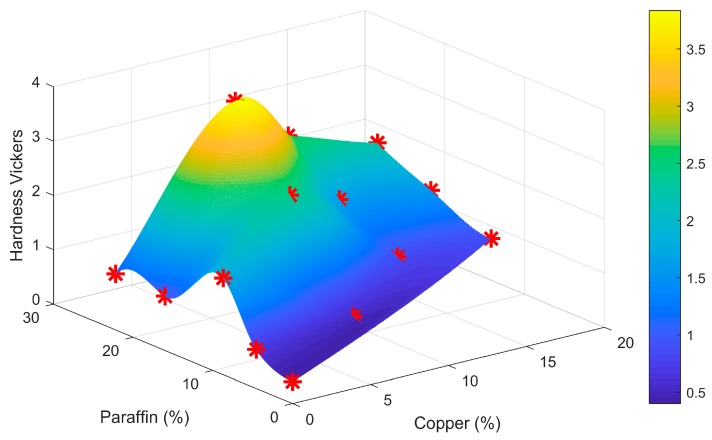
Vickers indentation test results (Hardness Vickers, vertical axis) for different composites (%Cu and Paraffin wax on bottom plane axis).

Figure 12 shows a point plot with a mesh created with cubic interpolation for hardness data versus weight percentage of paraffin and weight percentage of Cu. Although for the created curve, a maximum of 12% of Cu and 24% of paraffin weight content is observed for this approximation, the experimental data showed that the Cu increases the hardness values up to a certain extent (10 wt % approximately) and then exhibits a sharp decrease since the simultaneous addition of paraffin counters its effect.

The addition of a conductive phase has been attempted by others without incurring a significant loss in the heat of fusion [48,49,50]. For example, the combination of 1-octadecanol (stearyl alcohol) with graphene results in a PCM with a higher thermal conductivity (±140%) with a decrease of only 14% in the heat of fusion. The thermal properties of 1-octadecanol improve significantly with the addition of graphene, better than the effects of other nanofillers, such as silver nanowires or carbon nanotubes, reported previously in the literature [50]. Exfoliated graphite nanoplatelets (xGnP) can be added to paraffin waxes to create composite PCMs, achieving higher thermal conductivities, better thermal stabilities and similar latent heat [51]. xGnP can be embedded to a form-stable polyethylene glycol/polymethyl methacrylate composite PCM to enhance thermal and electrical conductivity, using the method of in situ polymerization upon ultrasonic irradiation [52]. Other authors prepared and investigated the properties of different kinds of paraffin/PUPCMs (polyurethane solid–solid PCMs), via bulk polymerization, with different mass fractions and obtained high enthalpies and broad phase transition temperature ranges [11]. In the case of the present research, the conductive phase seemed to improve the mechanical properties of the composites when contained in low percentages (<10 wt %), however, given their mass, the enthalpy (J/g) detected in the composites was reduced as the loading of the same increased. Recommendations for the future include the use of a lightweight, thermally conductive phase with nanosized particulates and higher aspect ratios that will allow percolation to take place at lower loadings.

In sum, despite obtaining low enthalpy values for the PCM-composite samples, this research provided a proof of concept that (a) a thickening agent is an indispensable filler to allow the incorporation of high loadings of PCMs and prevent dispersion issues, (b) organic PCMs are much more thermally stable than the inorganic ones employed, (c) epoxy matrices can act as the PCM reservoir, simplifying the fabrication process (since there is no need for encapsulation steps) and reducing production time and costs and (d) conductive phases (Cu particulates in this case) can improve the heat flow and mechanical stability of the composites.

## 4. Conclusions

This research successfully achieved the goal of producing novel formulations of PCM-epoxy composite systems containing a thickening agent and a thermally conductive phase. The results indicated that the epoxy matrix can act as container for the PCM phase without hindering the heat-absorbing behavior of the PCM employed. Organic PCMs presented reversible phase transformations over multiple cycles, an advantage that was lacking in their inorganic counterparts. The enthalpy values of the organic PCM-epoxy specimens increased linearly with the PCM content in the matrix. The use of thickening agents allowed the fabrication of specimens with up to 40% PCM loading without evident phase segregation, a loading significantly higher than others reported. In the composites, the epoxy resin acted as a supporting material which prevented the leakage of the melted paraffin. That is, these composite PCMs do not require a container for encapsulation, which lowers costs. The addition of a conductive phase produced an increase in the heat flow signal of the composites for all PCM weight percentages studied. Cu increased the composite samples hardness when present in small weight percentages (up to 10 wt %), but beyond that loading it had detrimental effects. Recommendations for the future include the use of a lightweight, thermally conductive phase with nanosized particulates and higher aspect ratios that will allow percolation to take place at lower loadings. The PCM-epoxy-thickening agent-conductive phase combination presents great potential as a heat-absorbing layer at the temperatures employed.

## Figures and Tables

**Table 1:** Advantages and disadvantages of different types of PCMs.

Type of PCM	Advantages	Disadvantages	Literature
Organic: Paraffins and non-paraffins	- High latent heat of fusion - Thermally and chemically stable - Freeze with little or no supercooling - Congruent phase change - Good nucleation rate - No segregation - Low vapor pressure in the melt form - Not dangerous - Nonreactive - Noncorrosive (except fatty acids) - Availability in a large temperature range - Recyclable	- Low thermal conductivity - Low volumetric latent heat storage capacity - Flammable - Noncompatible with plastic container	[13,18,19,20,21,22,23,24,25,26,27]
Inorganic: Hydrated salts	- Higher volumetric latent heat storage capacity - Higher latent heat of fusion - Higher thermal conductivity - Lower volume change - Sharper phase change - Nonflammable - Compatible with plastics - Lower manufacturing/disposal environmental impact - Low cost - Readily available	- High volume change - Supercooling problems - Poor nucleating properties (their application could require the use of some nucleating and thickening agents) - Incongruent melting and dehydration in the process of thermal cycling - Phase segregation during decomposition and phase separation - Corrosive to most metals - Slightly toxic	[13,18,19,20,22,23,24,25,26,27,28]
Eutectic	- Sharp melting temperature - Volumetric thermal storage density slightly above organic compounds - No segregation - Congruent phase change	- Limited data are available on their thermophysical properties	[13,18,24,29]

**Table 2:** Parameters description for Plackett-Burman method.

Parameter	Description	Levels		
		−	0	+
**A**	Amount of Cu *	5%	7.5%	10%
**B**	Amount of paraffin *	5%	7.5%	10%
**C**	Time to wait to add paraffin and Cu	10 min	15 min	20 min
**d1**	Dummy variable #1			
**d2**	Dummy variable #2			
**d3**	Dummy variable #3			
**d4**	Dummy variable #4			

* Weight percentage (of total).

**Table 3:** DSC experimental results for exothermic capacity for the inorganic PCMs used.

PCM	Exothermic Peak Temperature (°C)	Heat of Fusion (J/g)
PCM1 [Zn(NO_3_)_2_·4H_2_O]	40.50	70.96
PCM2 [Na_2_S_2_O_3_·5H_2_O]	70.20	251.10
PCM3 [NaCH_3_COO·3H_2_O]	68.50	356.50

**Table 4:** Plackett-Burman experimental design matrix and enthalpy results obtained from DSC.

Sample	A (% Cu)	d1	B (% Paraffin)	d2	C (Time 2)	d3	d4	Enthalpy
								(J/g)
#1	+	-	-	+	-	+	+	2.47
#2	+	+	-	-	+	-	+	4.88
#3	+	+	+	-	-	+	-	8.89
#4	-	+	+	+	-	-	+	8.31
#5	+	-	+	+	+	-	-	14.31
#6	-	+	-	+	+	+	-	3.82
#7	-	-	+	-	+	+	+	11.37
#8	-	-	-	-	-	-	-	2.76
#9	0	0	0	0	0	0	0	11.75
Effect	1.07	−1.25	7.24	0.25	2.99	−0.93	−0.69	*
SS	2.30	3.14	104.76	0.13	17.85	1.72	0.95	*
*F-value* **	1.55	*	70.66	*	12.04	*	*	*

Note: SS = Sum of squares; * no value; ** (*p* = 0.05). STD (±2% J/g).

**Table 5:** Samples developed to analyze paraffin and Cu influence on thermal behavior (STD (±2% J/g)).

% Cu	% Paraffin	Enthalpy (J/g)
10	5	2.47
10	10	8.89
10	20	15.32
10	30	22.06
10	40	25.98
20	40	24.60
30	40	23.81
